# Seroprevalence of *Leishmania* spp. Infection in Cats in Portugal

**DOI:** 10.3390/microorganisms14030668

**Published:** 2026-03-15

**Authors:** André Pereira, Joana Mourão, José Manuel Cristóvão, Ângela Xufre, Carla Maia

**Affiliations:** 1 Research in Veterinary Medicine, I-MVET, Faculty of Veterinary Medicine, Lusófona University-Lisbon University Centre, Campo Grande 376, 1749-024 Lisboa, Portugal; andre.duarte.pereira@ulusofona.pt (A.P.);; 2 Animal and Veterinary Research Center, CECAV, Faculty of Veterinary Medicine, Lusófona University-Lisbon University Centre, Campo Grande 376, 1749-024 Lisboa, Portugal; 3 Superior School of Health, Protection and Animal Welfare, ESPA, Polytechnic Institute of Lusophony, IPLUSO, Campo Grande, Rua do Telhal aos Olivais 8, 1950-396 Lisboa, Portugal; 4 Global Health and Tropical Medicine, GHTM, LA-REAL, Instituto de Higiene e Medicina Tropical, IHMT, Universidade NOVA de Lisboa, Rua da Junqueira 100, 1349-008 Lisboa, Portugal; 5 DNATech-Veterinary Clinical Analysis Laboratory, Estrada do Paço do Lumiar 22, 1649-038 Lisboa, Portugal; angela.xufre@dnatech.pt

**Keywords:** cats, *Leishmania*, One Health, seroprevalence

## Abstract

*Leishmania infantum* is endemic in Portugal, where dogs are the main reservoir and human visceral leishmaniosis remains a public health concern. Increasing evidence indicates that cats are also susceptible to infection and may contribute to local transmission, although nationwide data remain limited. This study aimed to estimate the seroprevalence of *Leishmania* spp. infection in cats in Portugal and to identify potential risk factors. Serum samples collected for unrelated clinical purposes and submitted to a veterinary diagnostic laboratory between December 2024 and March 2025, representing all districts of mainland Portugal and the Azores, were analyzed. Anti-*Leishmania* spp. antibodies were detected using the direct agglutination test, and associations with explanatory variables were evaluated through multivariable logistic regression. The overall seroprevalence was 8.9% (96/1080; 95%CI 7.3–10.7). One cat from Terceira Island (Azores) tested seropositive (1/10). Region was the only independent predictor of seropositivity, with cats from the Algarve showing higher odds of infection than those from other regions (adjusted OR 1.97; 95%CI 1.24–3.13; *p* = 0.004). These findings demonstrate widespread feline exposure consistent with known canine and human hotspots, and detection in the Azores suggests possible local transmission, supporting the inclusion of cats in multi-host surveillance within a One Health framework.

## 1. Introduction

*Leishmania infantum* is a protozoan parasite transmitted by phlebotomine sand flies and responsible for zoonotic leishmaniosis, a vector-borne disease of significant veterinary and public health concern in southern European countries, including Portugal [1]. Domestic dogs act as the primary reservoirs of the parasite, whereas humans are considered accidental hosts [2].

Climate and environmental changes are reshaping the distribution and abundance of sand fly vectors, increasing the risk of *Leishmania* infection in both animals and humans [3].

In Portugal, *L. infantum* infection is endemic [1]; accordingly, between 2010 and 2020, 214 autochthonous human cases of visceral leishmaniosis, the most severe form of the disease, were recorded in hospitals [4]. A recent nationwide survey of blood donors detected antibodies to *Leishmania* in 4.8% of participants, mainly from central regions of the country [5], confirming widespread exposure and ongoing transmission in the Portuguese population.

Canine infection is highly prevalent in Portugal [6]. The most recent nationwide survey, conducted in 2021, reported an overall *Leishmania* infection seroprevalence of 12.5%, exceeding 20% in several interior districts such as Castelo Branco and Portalegre [7]. This represents a marked increase compared with the 6.3% recorded in 2009 [8], suggesting intensified transmission and sustained exposure of domestic animals to competent sand fly vectors.

Although *L. infantum* infection in cats has long received less attention, growing evidence indicates that they are susceptible to infection and can develop clinical disease [9,10]. In endemic areas, cats are also frequently bitten by competent sand fly vectors [11]. Experimental and molecular studies demonstrate that infected cats can transmit *L. infantum* to sand flies [12] and harbor parasites genetically [13] and phenotypically [14] identical to those found in dogs, humans and vectors, indicating shared transmission cycles [15].

Regional studies in Portugal have reported feline *Leishmania* seroprevalence ranging from 0.0% to 3.7% [11,16,17,18,19,20,21,22]. A recent multicenter study conducted across the Mediterranean Basin countries reported a markedly higher prevalence of 24.7% in Portuguese cats, mainly among shelter and free-roaming populations [23]. In contrast, no seropositive cats have been detected to date in Madeira [16], and data from the Azores remain unavailable. This heterogeneity likely reflects differences in study design, diagnostic approaches, and feline population characteristics.

Therefore, this study aimed to estimate the seroprevalence of *Leishmania* spp. infection in cats in Portugal, using a similar methodological approach applied in a recent national canine serosurvey [7], and to investigate potential risk factors associated with feline exposure.

## 2. Materials and Methods

### 2.1. Study Design and Sampling

A nationwide cross-sectional seroepidemiological study was carried out in Portugal using feline serum samples collected in veterinary clinics between December 2024 and March 2025 and subsequently provided by a veterinary diagnostic laboratory (DNAtech, Lisbon, Portugal). All samples were collected for unrelated clinical purposes and anonymized before inclusion in the study.

The sample size (*n* = 1905) was calculated using EpiTools (https://epitools.ausvet.com.au, accessed on 20 December 2024), assuming a 95% confidence level, 3% precision, and an expected prevalence derived from a previous serosurvey conducted in dogs in mainland Portugal [7], adjusted to reflect the lower prevalence typically observed in cats from endemic regions [24] (Table S1, Supplementary Materials). For the Portuguese archipelagos of Madeira and the Azores, an expected prevalence of 0% was assumed [16]. At the time of study design, no autochthonous cases of feline or canine leishmaniosis had ever been reported.

A stratified random sampling strategy was applied to ensure nationwide representativeness. Feline population data from the *Sistema de Informação de Animais de Companhia* (SIAC), provided in May 2024, were used to define the proportional sample distribution across mainland districts and the islands of the Azores and Madeira. For each sample, available metadata included the district of origin, breed, age, and sex of the animal. Age groups were defined as kittens (<1 year), young (1–6 years), mature (7–10 years), and senior (>10 years) [23].

### 2.2. Detection of Anti-Leishmania spp. Antibodies

Anti-*Leishmania* spp. antibodies were detected using the direct agglutination test (DAT), as described by [20], with minor modifications. Briefly, feline sera were two-fold serially diluted from 1:25 to 1:51,200 in 0.9% NaCl containing 0.1 M β-mercaptoethanol. Subsequently, 50 µL of DAT antigen (1 × 10^7^ promastigotes/mL; KIT Biomedical Research, Amsterdam, The Netherlands) was added to each well. Plates were incubated at room temperature for 18 h and assessed visually. The antibody titer was defined as the reciprocal of the highest dilution showing clear agglutination, visible as large diffuse blue mats.

A serum dilution of 1:400 was used as the cut-off for positivity [7]. Each assay run included positive and negative controls to ensure test reliability and reproducibility. A serum sample from a cat with a confirmed IFAT titer of 1:1240 was used as the positive control [10], and a serum sample from a cat living in Switzerland, a non-endemic country, served as the negative control. Samples displaying ambiguous agglutination patterns were re-tested to ensure consistent interpretation.

### 2.3. Statistical Analysis

Descriptive and inferential analyses were performed using IBM^®^ SPSS^®^ Statistics v 25.0 (IBM Corp., Armonk, NY, USA). Age (in years) was summarized using the median and interquartile range (IQR), while categorical variables (i.e., region/district of origin, breed, age group, and sex) were expressed as counts and percentages. Seroprevalence was defined as the proportion of DAT-positive animals in the total sample, and 95% confidence intervals were calculated using Wilson’s method. Differences in seroprevalence across categories of the explanatory variables were assessed using Pearson’s chi-square test (χ2) or Fisher’s exact test when appropriate. Adjusted standardized residuals (ASR) were calculated, with values exceeding ± 1.96 considered to be indicative of statistically significant contributions at α = 0.05. DAT titers, originally expressed as two-fold serial dilutions, were transformed to log2 scale prior to analysis, and their correlation with age was assessed using the Spearman rank correlation coefficient (*rs*).

Variables with *p* ≤ 0.20 in univariable analyses were considered for inclusion in a logistic regression model, and results were reported as adjusted odds ratios (aOR) with 95% confidence intervals. The overall significance of the logistic regression model was assessed using the likelihood-ratio (*G*^2^) test, with *p* < 0.05 considered statistically significant.

A choropleth map illustrating district-level seroprevalence was produced in QGIS v3.44.4-Solothurn (QGIS Development Team, Open Source Geospatial Foundation, Beaverton, OR, USA). The district vector layer used for mapping was obtained from the *Sistema Nacional de Informação Geográfica* (SNIG; https://snig.dgterritorio.gov.pt, accessed on 2 November 2025).

## 3. Results

A total of 1080 cats were screened, with samples obtained from all districts of mainland Portugal and from the Azores islands of São Miguel e Terceira. No samples were available from the Madeira archipelago. District-level sampling coverage varied substantially across mainland Portugal. Eight districts (Porto, Aveiro, Leiria, Viseu, Lisboa, Santarém, Setúbal and Faro) reached at least 50% of the minimum required sample size (Table S1, Supplementary Materials).

The median age of sampled cats was 9 years (IQR: 4–13), and 54.5% (589/1080) were male. Most animals (910/994; 91.5%) were classified as European Shorthair.

Overall, 8.9% (96/1080) of cats tested seropositive for *Leishmania* spp., with antibody titers ranging from 1:400 to 1:102,400. Among seropositive cats, 5 (5.2%) showed titers of 1:400–1:800, 40 (41.7%) showed titers of 1:1600–1:6400, and 51 (53.1%) showed titers >1:6400. Seroprevalence differed significantly between regions (*p* = 0.007) (Table 1).

In mainland Portugal, the highest proportions were observed in the Algarve (36/248, 14.5%), followed by the North (19/191; 9.9%), whereas lower proportions were recorded in the Centre (11/226; 4.9%), and the Alentejo (1/34, 2.9%).

**Figure 1:**
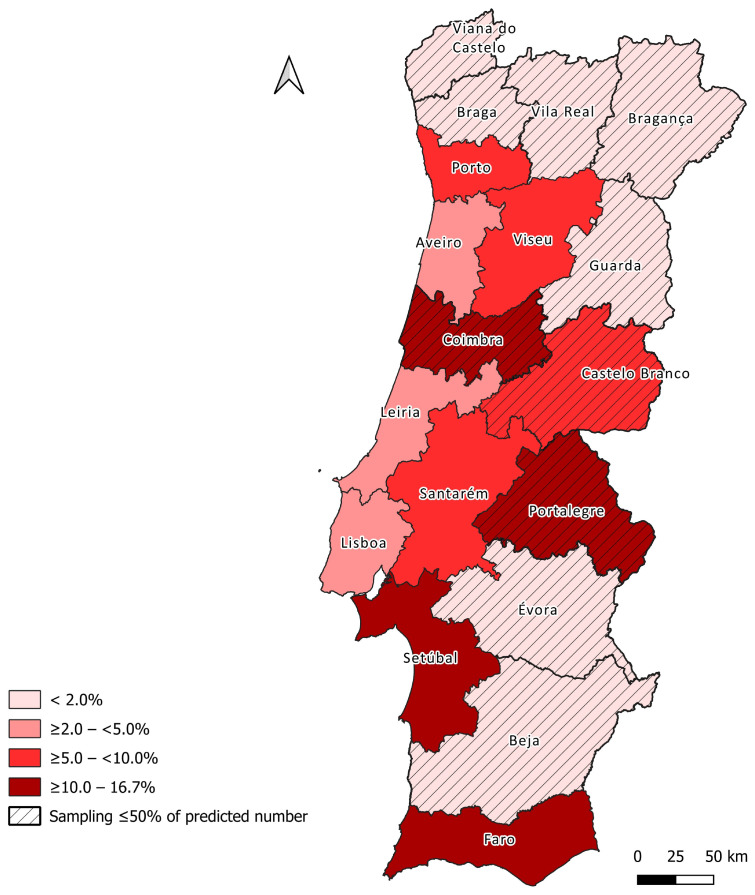
Estimated seroprevalence of *Leishmania* spp. infection in cats by district in mainland Portugal.

At district level, seroprevalence ranged from 0.0% to 16.7%, with the highest proportions recorded in Setúbal, Portalegre and Faro (Figure 1).

In the Azores archipelago, one seropositive cat from Terceira Island was identified (1/10; 10.0%).

No significant differences in seropositivity were found between sexes (*p* = 1.000), age groups (*p* = 0.249), or breeds (*p* = 0.369). In addition, no significant correlation was observed between age and antibody titers (*p* = 0.057).

In the multivariable logistic regression analysis, region was the only variable that remained significantly associated with *Leishmania* spp. seropositivity (*p* < 0.001). Cats from the Algarve showed higher odds of being seropositive compared with those from all other regions (aOR = 1.97; 95% CI: 1.24–3.13; *p* = 0.004).

## 4. Discussion

This nationwide survey provides the most comprehensive and geographically extensive evaluation of *Leishmania* spp. exposure in domestic cats conducted in Portugal, offering an updated view of feline infection pressure and relevant insight into the epidemiology of zoonotic leishmaniosis in the country.

The overall seroprevalence of 8.9% falls within the range reported for owned cats in Mediterranean countries, including Spain (7.2%), France (12.7%) and Italy (11.0%) [23], reinforcing that cats living in endemic areas are frequently exposed to infected sand flies. In the Portuguese context, the prevalence estimated in this study is higher than that reported in most feline serosurveys conducted to date [11,16,17,18,19,20,21,22]. However, previous studies were generally based on geographically limited or convenience-sampled populations. In addition, serological methods and cut-offs varied across studies, limiting representativeness and comparability. In contrast, the substantially higher prevalence reported by [23] for Portuguese cats reflects a distinct epidemiological picture dominated by shelter/free-roaming animals, populations known to experience greater risk of vector exposure and lower likelihood of preventive intervention [15,25,26]. Together, these observations indicate that feline exposure in Portugal is heterogeneous and influenced by population characteristics and diagnostic approaches and should therefore be interpreted in the context of each study’s design.

A meaningful understanding of feline exposure also benefits from comparison with canine data, given the established role of dogs as the primary domestic reservoir of *L. infantum* [27]. The most recent nationwide canine serosurvey reported a seroprevalence of 12.5% [7]. As this study follows an identical methodological framework, including sample size calculation, proportional stratified sampling, and the use of DAT with the same cut-off to detect anti-*Leishmania* antibodies, the comparison between species is epidemiologically robust. The proportion of seropositive cats observed here corresponds to approximately two-thirds of that reported in dogs in Portugal [7], following the pattern described by [24], in which feline exposure typically represents a substantial, though consistently lower, fraction of canine infection pressure. These findings support the view that, although cats are less frequently affected than dogs, they are nonetheless regularly exposed to the parasite and may contribute to local transmission dynamics [28,29].

Marked geographic variation in feline seroprevalence was evident across mainland Portugal. The highest value was observed in the Algarve, a region historically recognized for intense transmission and favorable conditions for phlebotomine sand flies [30]. At the district-level, the higher seroprevalences identified in Setúbal, Portalegre and Faro closely mirror known canine [7,8] and human hotspots [4,5], suggesting that cats are exposed within the same ecological and entomological landscape that sustains *Leishmania* spp. transmission in other hosts. The seroprevalence observed in Porto is also noteworthy. Although this district has traditionally been regarded as having lower risk, recent canine and human data have indicated increasing exposure in coastal northern districts [5,7]. The present findings are consistent with this pattern and highlight the importance of maintaining surveillance in regions where climatic suitability for sand flies may be expanding.

One of the most relevant findings of this study is the detection of anti-*Leishmania* spp. antibodies in a cat on Terceira Island, representing the first serological indication of feline exposure in the Azores. Although the autochthonous nature of the infection cannot be confirmed due to the lack of clinical or travel data, this observation gains epidemiological relevance given the recent description of a canine case in the archipelago [31]. Taken together, these findings suggest that the possibility of local transmission in the Azores merits further investigation, including targeted entomological surveys and broader host-based screening. In contrast, the absence of samples analyzed from Madeira represents a limitation. Nevertheless, a previous DAT-based survey in Madeira did not detect feline exposure [16], although updated data are needed to clarify whether autochthonous transmission is absent or simply under-detected.

No associations were observed between seropositivity and sex, age group or breed, a finding consistent with previous studies [11,32,33]. Most samples were obtained from cats classified as European Shorthair, a breed formally recognized by the *Fédération Internationale Féline* [34]. However, in clinical and epidemiological contexts this designation is often used to describe domestic short-haired cats in general, which may limit the ability to detect true breed-specific differences in seropositivity. The lack of age-related seropositivity suggests that cats of all ages are exposed to infection, underscoring the importance of prevention strategies that also must include juvenile animals. These may encompass several approaches to reducing vector exposure [28], such as the use of topical chemoprophylaxis (e.g., a matrix collar containing 10% imidacloprid and 4.5% flumethrin) [25]. More broadly, the strong regional effect observed in multivariable analysis reinforces that environmental and behavioral factors, rather than intrinsic host characteristics, are the primary determinants of exposure [28]. Future nationwide studies incorporating detailed lifestyle information, such as outdoor access, and the use of sand fly repellents, would help refine understanding of individual-level risk.

Several methodological considerations should be acknowledged. District-level sampling targets were not reached in all areas, limiting the ability to formally extrapolate local estimates to district populations. Future investigations specifically targeting these under-represented districts are needed to more accurately characterize the magnitude of local exposure. All samples were collected during the typical vector non-activity season [35], which minimizes the confounding effect of recent infections and provides a more stable reflection of cumulative exposure from the preceding transmission season [36]. The DAT cut-off of 1:400 was selected to ensure methodological comparability with the national canine survey [7]. Although DAT has been used in previous feline serosurveys [16,18,20,37], its analytical performance in detecting anti-*Leishmania* antibodies in cats remains insufficiently characterized. Moreover, no universally accepted serological reference standard has been established for feline epidemiological screening. Consequently, the absence of validated sensitivity and specificity estimates, together with the lack of species-specific data on potential cross-reactivity with other blood-borne pathogens, constitutes a methodological limitation and restricts the interpretation of serological results in feline populations. Nevertheless, Cardoso et al. [18] reported a high agreement between DAT and ELISA in cats, suggesting that DAT may still be informative for exposure assessment despite the lack of feline-specific validation data.

Among seropositive cats, antibody titers ranged from 1:400 to 1:102,400. However, titer magnitude should be interpreted with caution, as serological levels alone do not reliably distinguish active infection from prior exposure. Furthermore, molecular testing was not performed, given that only anonymized residual serum samples were available, and ongoing infection could therefore not be confirmed. The present findings should therefore be regarded as evidence of exposure rather than proof of active infection.

Clinical information was likewise unavailable, precluding any assessment of the relationship between seropositivity and compatible clinical manifestations. This is particularly relevant in feline leishmaniosis, as exposed cats may remain clinically inapparent and seropositivity should not be regarded as synonymous with disease. Information on feline retroviral co-infections, feline immunodeficiency virus (FIV) and feline leukemia virus (FeLV), was also unavailable. As these infections may influence immune status, susceptibility to *Leishmania* infection [38], antibody responses, and the clinical expression of feline leishmaniosis, the epidemiological and clinical significance of seropositivity in the present population should be interpreted with caution. From a clinical standpoint, the diagnosis of feline leishmaniosis should be based on an integrated interpretation of clinical, epidemiological, and laboratory findings [28]. Nevertheless, the prevalence observed in this survey indicates that feline exposure to *Leishmania* spp. is not negligible. Veterinarians working in endemic regions, including Portugal, should remain aware of this infection when evaluating cats presenting with compatible clinical manifestations, most commonly dermatological signs [28].

## 5. Conclusions

This study provides the most comprehensive national estimate of *Leishmania* spp. seroprevalence in domestic cats in Portugal, revealing an overall prevalence of 8.9% with marked geographic variation that mirrors recognized canine and human hotspots. From a clinical perspective, these findings highlight the need for increased awareness among veterinarians working in endemic areas, recognizing that seropositivity in cats primarily reflects exposure and should be interpreted within the epidemiological and clinical context. The first serological evidence of feline exposure in the Azores also raises the possibility that transmission may be occurring within the archipelago. These findings underscore the epidemiological relevance of cats and support their inclusion in coordinated surveillance efforts. In addition, the higher exposure detected in specific regions reinforces the importance of monitoring and managing stray and free-roaming cat populations at the municipal level, particularly in areas with known canine and human leishmaniosis, as part of integrated control strategies. Advancing a One Health approach will require integrated monitoring across animal, human and vector populations, strengthened diagnostic capacity, and focused entomological investigations to better delineate transmission pathways and guide evidence-based clinical and vector-control interventions.

## Figures and Tables

**Table 1:** Seroprevalence of *Leishmania* spp. infection in domestic cats according to host characteristics and geographical region.

*Variable/Categories*	*Tested*	*Seropositive*	*95% CI*	*p-Value/ASR*
Sex, *n* (%)	1080			*p* = 1.000
Female	491 (45.5)	44 (9.0)	6.7–11.8	0.1
Male	589 (54.5)	52 (8.8)	6.8–11.4	−0.1
Age (years), median (IQR)	9 (4–13)	7 (3–12)	NA	*p* = 0.057
Age group, *n* (%)	998			*p* = 0.249
Kitten	89 (8.9)	12 (13.5)	7.9–22.1	1.5
Young	295 (29.6)	27 (9.2)	6.4–12.0	0.1
Mature	191 (19.1)	20 (10.5)	6.9–15.6	0.8
Senior	423 (42.4)	31 (7.3)	5.2–10.2	−1.6
Breed, *n* (%)	994			*p* = 0.369
European Shorthair	910 (91.5)	82 (9.0)	7.3–11.0	
Others	84 (8.5)	6 (7.1)	3.3–14.7	
Region, *n* (%)	1080			*p* = 0.007
Azores	10 (0.9)	1 (10.0)	1.8–40.4	0.1
North	191 (17.7)	19 (9.9)	6.5–15.0	0.6
Centre	226 (20.9)	11 (4.9)	2.7–8.5	−2.4
Lisbon and Tagus Valley	371 (34.4)	28 (7.5)	5.3–10.7	−1.1
Alentejo	34 (3.1)	1 (2.9)	0.5–14.9	−1.2
Algarve	248 (23.0)	36 (14.5)	10.7–19.4	3.5
Total, *n* (%)	1080	96 (8.9)	7.3–10.7	

ASR: adjusted standardized residuals; CI: confidence interval; NA: not applicable.

## Data Availability

The datasets used and analyzed during the current study were provided by DNAtech and are not publicly available due to a confidentiality agreement. Data are, however, available from the corresponding author upon reasonable request and with permission from DNAtech.

## Supplementary Materials

The following supporting information can be downloaded at: https://www.mdpi.com/article/10.3390/microorganisms14030668/s1, Table S1: Sampling design and observed seroprevalence of *Leishmania* spp. infection in domestic cats by region and district.

## Abbreviations

The following abbreviations are used in this manuscript:

aOR	Adjusted odds ratio
ASR	Adjusted standardized residuals
CI	Confidence interval
DAT	Direct agglutination test
FeLV	Feline leukemia virus
FIV	Feline immunodeficiency virus
IFAT	Indirect Fluorescent Antibody Test
IQR	Interquartile range
OR	Odds ratio
SIAC	*Sistema de Informação de Animais de Companhia*
SNIG	*Sistema Nacional de Informação Geográfica*
