# Supplementary material for: Seroprevalence of Leishmania spp. Infection in Cats in Portugal
Source: Microorganisms. 2026 Mar 15;14(3):668. doi: 10.3390/microorganisms14030668 (PMC13029042; doi:10.3390/microorganisms14030668)
Supplement: Supplementary file 1 [file microorganisms-14-00668-s001.zip › microorganisms-4169062-supplementary.pdf]

Table S1. Sampling design and observed seroprevalence of *Leishmania* spp. infection in domestic cats by region and district.

Region (District)[1]	Expected prevalence (%)[2]	Feline population[3]	Required sample size[4]	Samples tested	Sampling coverage (%)[5]	Observed prevalence (%, IC95%)[6]
**North**	7.1	217947	283	191	67.5	9.9 (6.5-15.0)
Braga		47849	62	2	3.2	0.0 (0.0-65.8)
Bragança		6584	9	1	11.1	0.0 (0.0-79.3)
Porto		137422	181	183	101.1	10.4 (6.7-15.6)
Viana do Castelo		14605	19	4	21.1	0.0 (0.0-49.0)
Vila Real		9487	12	1	8.3	0.0 (0.0-79.3)
**Centre**	9.3	150951	360	226	62.8	4.9 (2.7-8.5)
Aveiro		47055	112	78	69.6	2.6 (0.1-8.9)
Castelo Branco		11626	28	12	42.9	8.3 (1.5-35.4)
Coimbra		32507	78	22	28.2	13.6 (0.5-33.3)
Guarda		6623	16	1	6.3	0.0 (0.0-79.3)
Leiria		32545	78	88	112.8	3.4 (1.2-9.5)
Viseu		20595	49	25	51.0	8.0 (0.2-25.0)
**Lisbon and Tagus Valley**	8.3	280758	326	371	113.8	7.5 (5.3-10.7)
Lisboa		178596	207	210	101.4	3.8 (1.9-7.3)
Santarém		30509	35	77	220.0	7.8 (3.6-16.0)
Setúbal		71653	83	84	101.2	16.7 (10.2-26.1)
**Alentejo**	12.0	27280	450	34	7.6	2.9 (0.5-14.9)
Beja		6635	109	9	8.3	0.0 (0.0-29.9)
Évora		15475	255	18	7.1	0.0 (0.0-17.6)
Portalegre		5170	85	7	8.2	14.3 (2.6-51.3)
Algarve	12.8	48809	476	248	52.1	14.5 (10.7-19.4)
Faro		48809	476	248	52.1	14.5 (10.7-19.4)
**Azores archipelago**	0.1	27953	5	10	200.0	10.0 (1.8-40.4)
São Miguel		15472	3	6	200.0	0.0 (0.0-39.0)
Terceira		5729	1	4	400.0	25.0 (4.6-69.9)
Graciosa		476	0	NA	NA	NA
São Jorge		419	0	NA	NA	NA
Pico		1958	0	NA	NA	NA
Faial		3108	1	0	0	NA
Flores		699	0	NA	NA	NA

Corvo		93	0	NA	NA	NA
Madeira archipelago	0.1	36226	5	0	0	NA
Madeira		3351	5	0	0	NA
Porto Santo		1693	0	NA	NA	NA
Santa Maria		1182	0	NA	NA	NA

[1] In mainland Portugal, geographical categories correspond to districts, in the Azores and Madeira archipelagos categories correspond to islands;[2] Expected prevalence reflects the expected regional prevalence used to derive the required sample size and was based on values from the national canine serosurvey [7], adjusted according to the feline–canine prevalence ratio described for endemic Mediterranean settings [24];[3] Feline population estimates correspond to the *Sistema de Informação de Animais de Companhia* (SIAC) data (May 2024);[4] Required sample size was calculated assuming a 95% confidence level, 3% precision and region-specific estimated prevalence, with district-level sample targets allocated proportionally to the feline population within each region;[5] Sampling coverage represents the proportion of the required sample obtained in each geographical unit; [6] Observed prevalence corresponds to the proportion of direct agglutination test-positive animals among those tested, with 95% confidence intervals calculated using Wilson's method; NA: not applicable.
